# Gastrointestinal parasites in young dogs and risk factors associated with infection

**DOI:** 10.1007/s00436-022-07760-9

**Published:** 2022-12-22

**Authors:** Lea-Christina Murnik, Arwid Daugschies, Cora Delling

**Affiliations:** Institute of Parasitology, Center for Infectious Disease, Faculty of Veterinary Medicine, Leipzig University, An Den Tierkliniken 35, 04103 Leipzig, Germany

**Keywords:** *Giardia*, Endoparasites, Dog, Germany, Zoonotic disease, Prevalence

## Abstract

Young dogs are particularly susceptible to infections with endoparasites. The occurrence of endoparasites was investigated in young dogs from Central Germany between July 2020 and July 2022. In total, 386 fecal samples originating from 171 dogs were examined for the prevalence of endoparasites using a combined flotation- and sedimentation technique and conventional PCR. Overall, in 41.2% (159/386) of the examined samples, endoparasites were detected. The most frequently occurring endoparasites were *Giardia duodenalis* (29%), *Cryptosporidium* spp. (9.1%), *Cystoisospora* spp. (7.3%), and *Toxocara canis* (6%). Sequencing of *G. duodenalis* positive samples showed that most infections belonged to the host-specific assemblages C (38.4% (43/112)) and D (35.7% (40/112)). The zoonotic assemblage A was identified in 8% (9/112) of the samples. Moreover, mixed infections were observed as follows: C/D in 5 (4.5%), D/A in 4 (3.6%), and C/A in 3 (2.7%) samples. All assemblage A infections were assigned to the potentially zoonotic subassemblage AI. Co-infections of *G. duodenalis* and *Cryptosporidium* spp. were observed in 3.1% (12/386) of the samples. Analyzing several host factors for their potential association with endoparasitic infection, the origin of dogs, as well as the living environment were identified as the main risk factors for infection with endoparasites. Overall, this study shows a high infection rate with endoparasites, especially *G. duodenalis*, in young dogs from Germany. The results of this study contribute to further insight into the distribution and potential risk factors associated with endoparasitic infections, as well as the zoonotic potential these parasites may present.

**Supplementary Information:**

The online version contains supplementary material available at 10.1007/s00436-022-07760-9.

## Background

Endoparasites rank among the most common causes of gastrointestinal disease in dogs. Young dogs (up to 1 year of age) are more often infected with endoparasites than older ones (Barutzki and Schaper 2003; Martínez-Carrasco et al. 2007; Becker et al. 2012; Ilić et al. 2021). Typical parasites found in dogs are *Cystoisospora* spp., *Toxocara canis*, *Toxascaris leonina*, hookworms (i.e., *Uncinaria* and *Ancyclostoma*), *Trichuris vulpis*, and the protozoon *G. duodenalis* (Barutzki and Schaper 2003; Deplazes 2006). *Cystoisospora* spp., *T. canis*, and *G. duodenalis* are highly prevalent and play an important role in the first year of the dog’s life since they are able to cause diarrhea and malabsorption, leading to retarded growth and weak puppies (Barutzki and Schaper 2013; Raza et al. 2018). Furthermore, eggs and oocysts of several endoparasites (e.g., *T. canis* and *Cystoisospora*) show a high tenacity to physical stress and may lead to intense contamination in a crowded environment (e.g., shelters) and therefore, to high parasite prevalence (Raza et al. 2018). Since *T. canis* can also be transmitted via the placenta or, less frequently, via breast milk, patent infection by this parasite may already be observed in the first weeks of the dogs’ life and can lead to severe disease (Gothe and Reichler 1990a; Schnieder et al. 2011; Deplazes et al. 2012).

*Giardia duodenalis* is a species complex consisting of eight assemblages A–H (Feng and Xiao 2011). The assemblages A and B are considered zoonotic since they have been found in humans as well as numerous mammals. These assemblages are further divided into subassemblages, consisting of three major groups (AI, AII, and AIII) within assemblage A and two main groups (BIII and BIV) within assemblage B. The subassemblage AI is considered zoonotic, the subassemblage BIII has been detected in dogs as well as humans, whereas subassemblages AII and BIV are assumed to be human specific. The subassemblage AIII has been almost exclusively found in wild ruminants (Traub et al. 2004; Feng and Xiao 2011; Cai et al. 2021). The remaining assemblages are assumed to have a narrower host range (Feng and Xiao 2011). Dogs are particularly affected by the canine-specific types C and D, which are responsible for most of the infections in dogs. However, in several studies, assemblages A and more rarely B have also been detected (Ballweber et al. 2010; Sotiriadou et al. 2013; Sommer et al. 2018; Uiterwijk et al. 2020). In addition to *G. duodenalis*, also other endoparasites, such as *T. canis*, are potentially zoonotic and transmitted via the fecal–oral route by direct contact with the agent or through contaminated water or food (Thompson and Monis 2012; Macpherson 2013; Chen et al. 2018). Because of the increasingly close contact between pets and humans, dogs attain growing attention as possible vectors for human pathogens (Overgaauw et al. 2020).

The aim of this study was to (1) investigate the prevalence of endoparasites in dogs during their first year of life, (2) identify potential risk factors associated with an endoparasitic infection, and (3) evaluate the possible zoonotic potential emanating from *G. duodenalis* positive samples.

## Material and methods

### Fecal samples

From July 2020 to July 2022, a total of 386 fecal samples were collected. The samples were obtained from 171 dogs, which were sampled up to three times during their first year of life. The study design has been described previously (Murnik et al. 2022). The dogs originated mainly from commercial breeders (*n* = 123), a few originated from animal shelters (*n* = 13) or private households (including dogs originating from animal protection organizations or other origins) (*n* = 35), from Central Germany (Saxony or Saxony-Anhalt). Briefly, the first fecal sample of each dog was usually collected at the age of about 8 weeks by the breeders. Six of these samples were sent in as a pooled litter sample, originating from 5 to 10 puppies. Afterward, the puppies were sold, and the new owners were asked to send in samples of the dogs with a time interval of 3–4 months in-between sampling. Depending on the age of the dogs at the time of sample submission, the samples were organized into 4 groups: 0–9 weeks, 10 weeks to 5 months, 6–9 months, and 10–12 months of age. The number of samples in each group was 129, 83, 123, and 51, respectively. Some of the owners missed one or more requested submissions despite repeated reminders for unknown reasons. Therefore, the number of samples in the different age groups differs from the overall number of participants.

Furthermore, the owners were asked to fill out a questionnaire concerning general information, e.g., gender, origin, living conditions, and clinical symptoms.

Fecal samples were collected on three consecutive days at each sampling date and shipped to the Institute of Parasitology (Faculty of Veterinary Medicine, Leipzig University Germany) for parasitological analysis. The samples were stored at 4 °C until further processing was conducted within 1–3 days.

### Detection of endoparasites

All fecal samples were examined for intestinal parasites by the combined flotation- and sedimentation technique. Briefly, an approximately apricot-sized amount of feces was mixed with water, filtered, and then left to sediment for 30 min. Of the resulting sediment, 1 ml was given into a 15-ml test tube and overlayered with NaNO_3_ (sodium nitrate and specific gravity of 1.3). After centrifugation for 5 min at 2000 rpm, the floated parasitic stages were examined by microscopy (Schmäschke 2013). A description of the laboratory examination of samples for *Cryptosporidium* spp. has already been published recently (Murnik et al. 2022) and will therefore not be addressed further here. For detecting *G. duodenalis*, all samples were analyzed using a conventional multi-locus PCR. DNA was extracted using the QIAamp® Fast DNA Stool Mini Kit (QIAGEN, Hilden, Germany) according to the manufacturer’s instructions with an initial ultrasonic treatment for 5 min. Purified DNA samples were stored at − 20 °C until further analysis was performed.

### PCR amplification of Giardia duodenalis

PCR amplifications were performed at three different gene loci to detect *G. duodenalis*. A semi-nested PCR protocol was used targeting the glutamate dehydrogenase gene (*gdh*) and a nested protocol for the genes beta-giardin (*bg*) and the small subunit ribosomal RNA (*ssurRNA*) gene. A detailed description of the PCR conditions and primers is listed in Table 1.

**Table 1:**
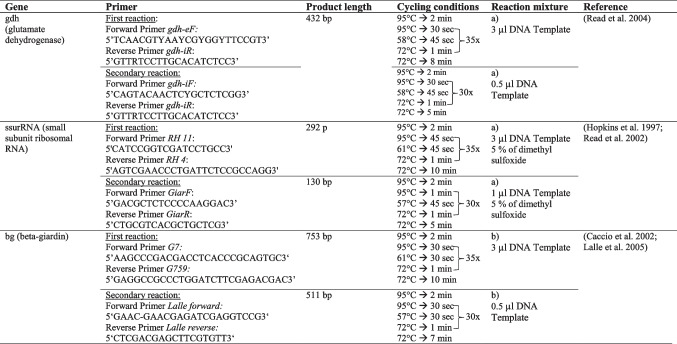
Primers and PCR conditions

a) Reaction mixture: 2.5 µl 10 × DreamTaq Buffer (Thermo Scientific ™), 0.8 µl NTPs, 0.5 µl forward primer (25 µM), 0.5 µl reverse primer (25 µM), 0.1 µl DreamTaq Green DNA Polymerase (Thermo Scientific™), and DEPC water to a total volume of 25 µl

b) Reaction mixture: 2.5 µl 10 × PCR Rxn Buffer (Invitrogen™), 0.8 µl NTPs, 0.5 µl forward primer (25 µM), 0.5 µl reverse primer (25 µM), 1.25 µl MgCl2 (50 mM) (Invitrogen™), 0.2 µl Platinum™ Taq DNA Polymerase (Invitrogen™), and DEPC water to a total volume of 25 µl

For each PCR reaction, a negative and positive control was included. The PCR products were analyzed by using a 1.5% agarose gel which was stained with ethidium bromide. Visualization of bands was performed by UV light.

### Sequencing

The positive PCR products of the secondary reaction were purified by using the PCR Purification Kit (Jena Bioscience GmbH, Jena, Germany) according to the manufacturer’s instructions. The purified DNA was stored at – 20 °C until sequencing was performed. Sequencing was conducted by Microsynth Seqlab (Göttingen, Germany) in both directions for all three genes. The resulting sequences were evaluated with MEGA version X. Consensus sequences from forward and reverse reads were created with the software BioEdit (version 7.2.5) and aligned to reference sequences from GenBank® using the Basic Local Alignment Search Tool (BLAST)https://blast.ncbi.nlm.nih.gov/Blast.cgi. Phylogenetic relationships were analyzed based on the *gdh* and *bg* genes. Multiple alignments were generated using MEGA version X, and two trees were constructed based on the maximum likelihood method (Tamura-Nei model).

### Data analysis

The obtained data was collected and analyzed using Microsoft Excel Version 16.63.1 (Microsoft Corporation, Redmond, USA). Statistical analysis was performed using SPSS statistics 27 (IBM, Armond, USA). Statistical results were considered significant if *p* < 0.05. Binary logistic regression was used to test associations between the infection status and different categories e.g., age, fecal consistency, and origin, whereby the outcome was the infection status (0 = no infection and 1 = infection). Unknown data was excluded from the analysis as missing values.

## Results

### Endoparasites

A total of 386 fecal samples were investigated for endoparasites, giving an overall prevalence of 41.2% (159/386). The intestinal parasites identified most often, besides *Giardia* (results are shown below) and *Cryptosporidium*, were *Cystoisospora* spp. and *T. canis,* with 7.3 (28/386) and 6% (23/386), respectively. *Cystoisospora* spp. was mostly found in the youngest age group (4.7%, 18/386), whereas the age groups of 10 weeks–5 months and 6–9 months were mostly probed positive for *T. canis* with 1.8 (7/386) and 2.8% (11/386), respectively. Further distribution of the endoparasites according to the different age groups is shown in Table 2.

**Table 2:** Distribution and prevalence of endoparasites considering the different age groups of dogs

Parasite	Number of samples (%)								Total (%)	
Age	0–9 weeks		10 weeks–5 months		6–9 months		10–12 months			
	Positive	Negative	Positive	Negative	Positive	Negative	Positive	Negative	Positive	Negative
Endoparasites total	30 (23.3)	99 (76.6)	46 (55.4)	37 (44.6)	59 (48.0)	64 (52.0)	24 (47.1)	27 (52.9)	159 (41.2)	227 (58.8)
*Toxocara canis*	1 (0.8)	128 (99.2)	7 (8.4)	76 (91.6)	11 (8.9)	112 (91.1)	4 (7.8)	47 (92.2)	23 (6.0)	363 (94.0)
*Cystoisospora* spp.	18 (14.0)	111 (86.0)	6 (7.2)	77 (92.8)	4 (3.3)	119 (96.7)	-	51 (100)	28 (7.3)	358 (92.7)
Taeniidae	-	129 (100)	-	83 (100)	2 (1.6)	121 (98.4)	1 (2.0)	50 (98.0)	3 (0.8)	383 (99.2)
*Toxascaris leonina*	-	129 (100)	-	83 (100)	1 (0.8)	122 (99.2)	1 (2.0)	50 (98.0)	2 (0.5)	384 (99.5)
Hookworms	-	129 (100)	-	83 (100)	-	123 (100)	1 (2.0)	50 (98.0)	1 (0.3)	385 (99.7)
*Capillaria spp.*	-	129 (100)	-	83 (100)	-	123 (100)	1 (2.0)	50 (98.0)	1 (0.3)	385 (99.7)
*Trichuris vulpis*	-	129 (100)	1 (1.2)	82 (98.8)	-	123 (100)	-	51 (100)	1 (0.3)	385 (99.7)
*Giardia duodenalis*	13 (10.1)	116 (89.9)	31 (37.3)	52 (62.7)	48 (39.0)	75 (61.0)	20 (39.2)	31 (60.8)	112 (29.0)	274 (71.0)
*Cryptosporidium* spp.	10 (7.8)	119 (92.2)	17 (20.5)	66 (79.5)	7 (5.7)	116 (94.3)	1 (2.0)	50 (98.0)	35 (9.1)	351 (90.9)

The majority of dogs (118/171) remained negative throughout the whole study period. Of the 171 dogs, 42 (24.6%) tested positive for parasite infection at one sampling point, and 10 (5.8%) dogs were probed positive two times. Only one (0.6%) dog was found to harbor endoparasites at all three sampling time points.

Although the majority of positive fecal samples were found in the age group 10 weeks–5 months (55.4% positive), no statistically significant association was found between the presence of endoparasites and the age of the dogs (Table 3).

**Table 3:** Binary logistic regression results for the presence of endoparasite infections

Variable	Categories	Endoparasites total (%)		Total	Odds ratio (95% confidence intervals)	*P*-value
		Positive	Negative			
Age group (*n* = 386)	0 to 9 weeks	30 (23.3)	99 (76.7)	129	Reference	
	10 weeks to 5 months	46 (55.4)	37 (44.6)	83	3.267 (0.824–12.961)	0.092
	6 to 9 months	59 (48.0)	64 (52.0)	123	1.577 (0.368–6.757)	0.540
	10 to 12 months	24 (47.1)	27 (52.9)	51	2.398 (0.540–10.635)	0.250
Sex	Male	66 (36.3)	116 (63.7)	182	Reference	
	Female	88 (44.4)	110 (55.6)	198	1.479 (0.850–2.572)	0.166
Origin	Breeder	133 (38.3)	214 (61.7)	347	Reference	
	Shelter	13 (65.0)	7 (35.0)	20	2.988 (1.163–7.680)	0.023*
	Animal protection organization	7 (58.3)	5 (41.7)	12	2.253 (0.701–7.242)	0.173
	Others	4 (80.0)	1 (20.0)	5	6.436 (0.712–58.200)	0.097
Residency	Rural areas	75 (33.3)	150 (66.7)	225	Reference	
	Small town	22 (47.8)	24 (52.2)	46	1.949 (0.732–5.185)	0.181
	Big city	62 (53.9)	53 (46.1)	115	2.198 (1.123–4.301)	0.022*
Contact to other dogs	Permanently	59 (35.8)	106 (64.2)	165	Reference	
	Regularly	86 (48.0)	93 (52.0)	179	0.625 (0.255–1.531)	0.304
	Rarely	13 (34.2)	25 (65.8)	38	0.488 (0.133–1.787)	0.279
	Never	1 (25.0)	3 (75.0)	4	0.000	0.999
Gastrointestinal symptoms at the timepoint of sampling	Yes	8 (28.6)	20 (71.4)	28	Reference	
	No	151 (42.2)	207 (57.8)	358	0.739 (0.082–6.671)	0.788
Antiparasitic treatment	Yes	108 (39.1)	168 (60.9)	276	Reference	
	No	40 (48.8)	42 (51.2)	82	2.511 (0.773–8.157)	0.126

* = statistical significance

Most fecal samples originated from breeders (*n* = 347), and only a few from shelters (*n* = 20), imported from animal protection organizations (*n* = 12), or other origins (*n* = 5). The origin “breeders” was defined as the reference, and a significant association between the infection status and the origin was determined for dogs originating from shelters (*p* = 0.023) (Table 3).

Dogs living in big cities (e.g., Leipzig, Dresden; > 500,000 citizens) were significantly more often infected with intestinal parasites (53.9% positive; *p* = 0.022) (Table 3).

No statistically significant association between the presence of endoparasites and contact with other dogs, the gender, as well as the presence of gastrointestinal symptoms, and the reported performance of standard antiparasitic treatment (every 3–4 months) could be determined (Table 3).

### Giardia duodenalis

Of the 386 fecal samples probed, 29% (112/386) tested positive for *G. duodenalis.* Therefore, *G. duodenalis* was the most prevalent parasite in the examined samples. The majority of fecal samples showed a firm (*n* = 245) or soft (*n* = 115) consistency. No statistically significant association between the infection status and the fecal consistency could be determined (Table 4).

**Table 4:** Binary logistic regression results for the presence of *Giardia duodenalis* in association with age and fecal consistency

Variable	Categories	*Giardia duodenalis* infection (%)		Total	Odds ratio (95% confidence intervals)	*P*-value
		Positive	Negative			
Age group (*n* = 386)	0 to 9 weeks	13 (10.1)	116 (89.9)	129	Reference	
	10 weeks to 5 months	31 (37.3)	52 (62.7)	83	6.493 (3.037–13.879)	< 0.001*
	6 to 9 months	48 (39.0)	75 (61.0)	123	6.893 (3.370–14.098)	< 0.001*
	10 to 12 months	20 (39.2)	31 (60.8)	51	7.234 (3.123–16.756)	< 0.001*
Fecal consistency (*n* = 386)	1 (firm)	73 (29.8)	172 (70.2)	245	Reference	
	2 (soft)	33 (28.7)	82 (71.3)	115	1.695 (0.975–2.948)	0.061
	3 (mushy)	6 (25.0)	18 (75.0)	24	1.332 (0.466–3.803)	0.593
	4 (watery)	-	2 (100)	2	0.000	0.999
	5 (bloody)	-	-	-	-	-

* = statistical significance

The majority of dogs (84/171, 49.1%) were negative for *Giardia* throughout the whole sampling period, whereas 56 dogs (32.8%) tested positive once. Furthermore, in 24 dogs (14%) *Giardia* could be found on two occasions, and in 7 dogs (4.1%), all three fecal samples tested positive within the sampling period.

Dogs of the age groups 10 weeks to 5 months, 6 to 9 months, and 10 to 12 months were more frequently positive for *Giardia* spp. than those of the younger reference group (0 to 9 weeks) (Table 4).

Sequence analysis revealed that the majority of the *Giardia-*positive samples belonged to the canine-specific assemblages C (38.4%) and D (35.7%). Furthermore, assemblage A was found in 9 (8%) of the *Giardia-*positive samples. Moreover, mixed infections with different assemblages were found in 12 of the specimens. The mixed infections were identified as follows: C/D in 5 (4.5%), D/A in 4 (3.6%), and C/A in 3 (2.7%) of the samples. Regarding eight of the examined samples, the sequence analysis was not successful. Multi-locus genotyping results are presented in the additional file (Table S1).

**Fig. 1:**
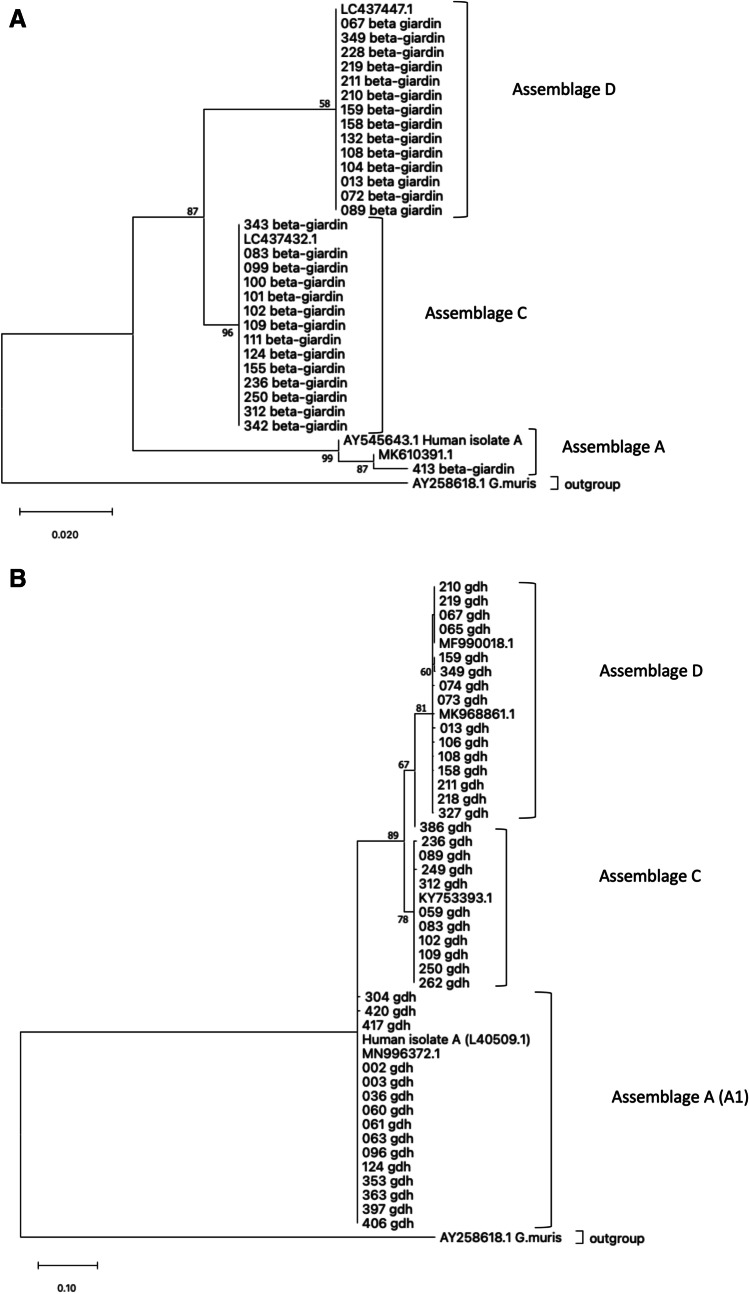
Maximum likelihood tree (Tamura-Nei model) showing the phylogenetic relationship of the *beta-giardin* (**A**) and the *gdh* (**B**) gene. The numbers on branches indicate the percent bootstrapping values over 50% by using 1000 replicates. The sequence of *G. muris* was used as an outgroup in each tree

Of the nine assemblages A positive samples, only two were successfully amplified at the *ssurRNA* and *gdh* gene loci. Six were only successfully amplified at the *gdh* gene locus, and one was successfully amplified at the *bg* gene locus. The seven mixed infections containing assemblage A among others were also successfully amplified at the *gdh* gene locus. All of these samples were assigned to the subassemblage AI (Fig. 1). Three of the herein obtained sequences were deposited in GenBank® (OP562097–OP562099).

### Coinfections

Mixed infections could be detected in 10.6% (41/386) of all samples. As described previously (Murnik et al. 2022), *Cryptosporidium* spp. was found frequently in 9.1% (35/386) of the examined samples. Interestingly, a coinfection of *G. duodenalis* and *Cryptosporidium* spp. could be identified in 3.1% (12/386) of the samples. Therefore, the most frequently detected mixed infection contained these two protozoa *G. duodenalis* and *Cryptosporidium* spp., with 29.3% (12/41). This was followed by mixed infections of *G. duodenalis* and either *T. canis* or *Cystoisospora* spp., in both cases with a prevalence of 19.5% (8/41), which is shown in Table 5.

**Table 5:** Coinfections of endoparasites

Parasites detected	Number of cases	Percentage of coinfection (%)
*G. duodenalis* + *Cryptosporidium* spp.	12	29.3
*G. duodenalis* + *T. canis*	8	19.5
*G. duodenalis* + *Cystoisospora* spp.	8	19.5
*G. duodenalis* + *Taeniidae*	1	2.4
*G. duodenalis* + *Cryptosporidium* spp. + *Cystoisospora* spp.	2	4.9
*G. duodenalis* + *Cryptosporidium* spp. + *T. canis*	1	2.4
*G. duodenalis* + *Taeniidae* + *T. canis*	2	4.9
*G. duodenalis* + *Cystoisospora* spp. + *T. canis*	1	2.4
Total	41	

## Discussion

The overall prevalence of endoparasites in the herein-examined samples was 41.2% (159/386). This is similar to other studies conducted in Germany, where slightly lower prevalence rates of 32.2 and 30.4% were found in two large-sized studies with 8438 and 24,677 dog samples, respectively. The fecal samples mentioned above were sent in for diagnostic purposes to the laboratory for various reasons (Barutzki and Schaper 2003, 2011). In contrast, a strikingly low prevalence of 9.4% was found by examining 445 stray and foster dogs in Lower Saxony (Germany) (Becker et al. 2012). Similar to our findings the most frequently found parasites in these studies, besides *Giardia,* were *T. canis* and *Cystoisospora* spp. (Barutzki and Schaper 2003, 2011; Ortuño and Catella 2011; Becker et al. 2012; Ilić et al. 2021).

*Toxocara canis* and *Cystoisospora* spp. are considered to be typical endoparasites in young dogs. *T. canis* is also assumed to be one of the most important gastrointestinal parasites in dogs of all ages (Schnieder et al. 2011; Becker et al. 2012). However, *T. canis* was the endoparasite that was detected by coproscopy as early as in the third week of the puppies’ life, followed by *Cystoisospora* spp. in the fourth week of life (Barutzki and Schaper 2013). We also found *T. canis* in the youngest age group (0–9 weeks); however, this group showed the lowest prevalence for *T. canis* of all age groups considered. *Cystoisospora* spp. plays a minor role in older dogs since the parasite induces immunity in older dogs (Schnieder 2006). This is in accordance with our results showing a decreasing prevalence of *Cystoisospora* spp. with increasing age of the dogs. Although *T. canis* and *Cystoisospora* spp. may cause severe disease (Lappin 2010; Schnieder et al. 2011; Raza et al. 2018), we did not find any association between gastrointestinal symptoms and the occurrence of endoparasites. This is in agreement with studies performed by other researchers showing an association between disease and lifestyle more frequent than a link to the occurrence of specific pathogens (Stavisky et al. 2011).

In one study conducted in Spain, *T. vulpis* was found quite frequently, with 11% of the samples being positive. In addition, hookworms were detected in a large percentage of dogs (16.9%) in Serbia (Ortuño and Catella 2011; Ilić et al. 2021). In our study, these two parasites were detected only in a very small fraction of the examined dogs, with one sample being positive for either of these parasites. Thus, the prevalence was low as expected with reference to former studies performed, and both roundworms appear not to deserve particular attention in Germany (Epe et al. 2004; Raue et al. 2017).

Cestodes of the family Taeniidae were only found in three (0.8%) of the examined samples, which is in accordance with another study from Germany where over a period of 10 years, only 0.4% (12/2731) of the examined samples were tested positive (Raue et al. 2017). In another long-term study from Germany, 1281 dog feces samples were examined, and 0.8% were found to be positive for taeniids (Epe et al. 2004). Although the prevalence is rather low, taeniid cestodes are diagnostically relevant since differentiation between non-zoonotic *Taenia* spp. and the zoonotic genus *Echinococcus* spp. by conventional microscopy of parasite eggs in dog feces is not possible.

All dogs positive for taeniid eggs were living in rural areas, indicating that contact with wildlife or livestock animals increases the risk of patent taeniid infection by dogs (Grandi et al. 2021; Waindok et al. 2021). This is supported by a previous study regarding wild gray wolves in Lower Saxony (Germany), where Taeniidae were found as the second most prevalent parasites (21.74%) (Bindke et al. 2019). In another study from northern Germany *Echinococcus* spp. (26.3%) was among the most prevalent parasites in red foxes and also *Taenia* spp. (16.3%) was detected frequently (Waindok et al. 2021). Therefore, especially concerning dogs living in rural environments, these parasites deserve attention. Furthermore, foxes increasingly invade urban areas, which is related to the risk of establishment of transmission cycles for cestodes with rodents serving as an intermediate host. However, our current data obtained from a limited number of animals or samples do not reflect such risk at present.

In one of the examined samples (0.3%), eggs of *Capillaria* spp. were found. *Capillaria* is not a frequently found parasite in dogs in Germany, which makes this observation quite interesting (Barutzki and Schaper 2003, 2011; Epe et al. 2004). However, the dog concerned lived on a farm with numerous other animal species in the same location, including poultry. Therefore, it appears possible that this finding rather reflects an intestinal passage of *Capillaria* eggs of poultry origin than a patent infection.

Previous studies have shown that dogs living in shelters, on the street, or in breeding facilities are more often infected with parasites than household dogs (Bugg et al. 1999; Palmer et al. 2008; Katagiri and Oliveira-Sequeira 2008; Mircean et al. 2017). This was confirmed in our study. Dogs originating from shelters were significantly more often infected with endoparasites than dogs of another origin (*p* = 0.023). However, it has to be kept in mind that the majority of the examined samples originated from breeding facilities and only a very small number from shelters or private households, which may cause some bias. Nevertheless, living conditions have an impact on the occurrence of parasites, and close contact between dogs in a confined and crowded environment seems to increase the prevalence of intestinal parasitism.

In contrast to a previous study by Kubas et al. (2022), in this study, the examined dogs living in urban environments were infected more often with endoparasites (*p* = 0.022) than the examined dogs from small towns or rural areas. This may be explained by a higher density of dogs in larger cities associated with more interaction between dogs and therefore, a higher risk of transmission due to direct contact or environmental contamination with excreted parasite stages.

No significant differences regarding the occurrence of infection with intestinal parasites were observed between female and male dogs in the present study. This finding is in accordance with previous studies, where also no association between infection status and gender were detected (Fontanarrosa et al. 2006; Becker et al. 2012).

In several studies, age was identified as a risk factor for the infection with endoparasites, reporting higher prevalence rates in dogs younger than 1 year of age (Martínez-Carrasco et al. 2007; Barutzki and Schaper 2011; Becker et al. 2012; Tamponi et al. 2017; Ilić et al. 2021). However, we could not find a significant association between the occurrence of endoparasites and the age of the examined dogs in our study. On the other hand, regarding exclusively the prevalence of the protozoon *G. duodenalis,* a significant difference compared to the reference age (0–9 weeks) was found (Table 4). Several other studies reported a higher risk of giardiasis for dogs younger than 1 year of age, especially within their first 6 months (Palmer et al. 2008; Batchelor et al. 2008; Gates and Nolan 2009; Barutzki and Schaper 2011; Itoh et al. 2015). In general, it seems to be reasonable to find high prevalence rates of endoparasites in young animals due to their immature immune system and potential stress factors associated with moving to a new home and weaning. Furthermore, transplacental or transmammary routes of infection for parasites such as *T. canis* have to be considered (Burke and Roberson 1985; Pereira et al. 2019).

The prevalence rate of *G. duodenalis* found in this study was 29% (112/386), which is similar to other studies conducted in Germany. In a study with 270 dogs from different animal shelters in Germany, 29.5% of them were found positive using a *Giardia* coproantigen ELISA (Cirak and Bauer 2004). Examining canine samples originating mainly from private households in different parts of Germany, similar results (30.6%) were described by Sommer et al. (2018). Instead, a slightly lower prevalence rate of 11.4% was found in 341 canine samples from stray and foster dogs in Lower Saxony (Becker et al. 2012). This is similar to the findings of Sotiriadou et al. (2013), who found a lower prevalence of 6.2% in 81 clinically suspicious dogs from Germany. Examining 2319 dog samples, Barutzki and Schaper (2013) found the highest prevalence rate of 52.5% positive for *Giardia* in 12 weeks old dogs. However, it has to be kept in mind that different age groups and various living conditions of the examined dogs, as well as the respective chosen diagnostic method, complicate the comparability of various studies.

Mixed infections were detected in a total of 41 (10.6%) of the examined samples. Twelve dogs (3.1%) were found to be positive for *G. duodenalis* as well as for *Cryptosporidium* spp. at the same sampling time point, which was the most common coinfection in this study. In another study from Germany, only one of 81 dogs harbored a mixed infection of *G. duodenalis* and *Cryptosporidium* (Sotiriadou et al. 2013). Similar observations were reported in northern Spain, where a coinfection was detected in 1.5% (3/194) of the dog fecal samples, and in China, where 13 of 485 dogs were coinfected (Xu et al. 2016; Gil et al. 2017). A higher coinfection prevalence was found in a study from Norway identifying both parasites in thirty-five dogs at the same sampling time points, as well as in a study from Madagascar, in which 15% of the examined dogs showed coinfection (Hamnes et al. 2007; Spencer and Irwin 2020). Mixed infections of *G. duodenalis* and *Cystoisospora* spp. (8/41), as well as *G. duodenalis* and *T. canis* (8/41), were frequently detected with 19.5% in each case. This is in accordance with another study from Germany where coinfection of *Cystoisospora* spp. and *G. duodenalis* was the most prevalent (28.0%), followed by a mixed infection of *Cystoisospora* spp. and *T. canis* (16.0%), and *T. canis* combined with *G. duodenalis* (12.0%) (Barutzki and Schaper 2013). Our results and those of previous studies confirm that coinfection with two or more parasites occur frequently in dogs. Data on the clinical relevance of coinfections, as well as information about the interaction between parasites, is lacking. However, in calves, it was shown that mixed infections were more often detected in diarrheic calves than in healthy ones and that diarrhea was more severe when mixed infections were present (de la Fuente et al. 1999; Brar et al. 2017). Such findings have not yet been reported in dogs and need further research.

In order to determine the predominant subassemblages of *G. duodenalis,* all fecal samples were examined by conventional multi-locus PCR. The multicopy gene locus *ssurRNA* revealed most of the *Giardia* positive samples compared to the two single gene loci *gdh* and *bg* (supplementary material table S1)*.* This is in accordance with previous studies, which also described striking differences between the respective gene loci (Dado et al. 2012; Sommer et al. 2015; Rehbein et al. 2019; Kim et al. 2019). This observation might be explained by the high sensitivity of the method or by multi-copy and conserved characteristics of the *ssurRNA* gene (Cacciò and Ryan 2008). Low parasite numbers in the fecal samples may also contribute to the differences since Adamska et al. (2010) described a detection limit of 100 cysts per 200 μl for the *bg* gene. Additionally, the DNA extraction method, the occurrence of possible PCR inhibitors as well as the different potential of the gene loci toward the assignment of assemblages may contribute to differences between the PCR results (Adamska et al. 2010; Feng and Xiao 2011; Thompson and Ash 2016; Rehbein et al. 2019). Although the *ssurRNA* gene was the most successful gene locus, the ability to identify subassemblages based on this gene is very limited (Thompson and Ash 2016; Hernández et al. 2021). Better information concerning the subassemblages of assemblages A and B are provided with the genes *gdh* and *bg*, and therefore, these two genes were used for the phylogenetic analysis (Fig. 1) (Thompson and Ash 2016; Hernández et al. 2021)*.* Unfortunately, in our study, we were not able to identify the subassemblage on more than one gene locus.

In accordance with former studies, genotyping results showed that the majority of the *Giardia* positive samples were allocated to the canine type, consisting of the assemblages C and D (38.4 (43/112) and 35.7% (40/112)), respectively (Johansen et al. 2014; Pallant et al. 2015; Zhang et al. 2017; Sommer et al. 2018; Uiterwijk et al. 2020). Furthermore, we were able to identify 8% (9/112) of the isolates as the potentially zoonotic assemblage A. Further analysis of 8 samples on the *gdh* gene and 1 sample on the *bg* gene locus showed the association with the subassamblage AI, indicating a potential zoonotic risk arising from these infected dogs since the subassemblage AI has been described to occur also in humans (Feng and Xiao 2011; Lecová et al. 2018)**.** Coinfection with multiple assemblages could be found in 12 samples as follows: 2.7% (3/112) as A and C, 3.6% (4/112) as A and D, and 4.5% (5/112) as C and D. These findings are in accordance with former studies in which coinfections were also detected (Leonhard et al. 2007; Dado et al. 2012; Johansen et al. 2014). Regarding eight of the examined samples (7.1%), we were not able to identify the associated assemblage.

Assemblage A is one of the most frequently found *Giardia* assemblages in humans (Feng and Xiao 2011). Especially in immunocompromised people or in humans living in poor regions in developing countries, *Giardia* is found regularly (Feng and Xiao 2011). Although considered to be specific to canids, *Giardia* of the assemblage C was detected in a symptomatic patient from Egypt who was undergoing immunosuppressive treatment (Soliman et al. 2011). This observation could be confirmed in further studies, which also detected the canid type in humans (Sprong et al. 2009; Broglia et al. 2013; Liu et al. 2014). In Germany, one of the main risk groups for giardiasis is children, with the highest average annual incidence rate within the age range of 1–5 years (11.5/100,000 on average) (Sagebiel et al. 2009). In the year 2020, the Robert-Koch-Institute recorded 2.2 cases of the disease for 100,000 persons within the age group 1–9 years, which was an incidence peak of *Giardia* infections in Germany (Robert-Koch-Institut 2020).

Although the presence of assemblage A in dogs and the finding of dog-specific assemblage C in humans indicates the possibility of zoonotic transmission for humans, however, the risk for zoonotic infection with *Giardia* in Germany is considered to be extremely low (Rehbein et al. 2019).

## Conclusion

The current results confirm that young dogs are often infected with endoparasites. Especially the protozoon *G. duodenalis* occurs frequently in young dogs from Central Germany, with 29% of the samples being positive in this study. The parasite prevalence rate underlines that further efforts to control parasite infection in dogs are justified, especially in crowded environments (e.g., shelters, breeders, and urban areas). Although potential zoonotic parasites were detected, the risk of infection and related disease is considered to be extremely low for immunocompetent humans in Germany. Coinfections of two or more parasite species seem to be common; however, further research on the impact on the clinical outcome, as well as potential interactions between parasites during those coinfections, is required.

## Supplementary Information

Below is the link to the electronic supplementary material.

Supplementary file1 (DOC 158 KB)

## Data Availability

The material obtained in this study is stored at the Institute of Parasitology, Faculty of Veterinary Medicine, Leipzig University. Representative nucleotide sequences obtained in this study were submitted to GenBank® under the accession numbers OP562097, OP562098, and OP562099.
