# Supplementary material for: Gastrointestinal parasites in young dogs and risk factors associated with infection
Source: Parasitol Res. 2022 Dec 22;122(2):585–96. doi: 10.1007/s00436-022-07760-9 (PMC9849189; doi:10.1007/s00436-022-07760-9)
Supplement: Supplementary file 1 — Supplementary file1 (DOC 158 KB) [file 436_2022_7760_MOESM1_ESM.doc]

**Gastrointestinal parasites in young dogs and risk factors associated with infection**

Lea-Christina Murnik, Arwid Daugschies, Cora Delling

Corresponding author: Lea-Christina Murnik
Email: [L.murnik@gmx.de](mailto:L.murnik@gmx.de)

**Journal:** Parasitology research

Table 1 Assemblages of Giardia duodenalis isolates from dogs at the ssurRNA, gdh and bg loci

| Sample-ID | *ssurRNA* | Accession Number | *gdh* | Accession Number | *bg* | Accession Number | Assemblage |
| --- | --- | --- | --- | --- | --- | --- | --- |
| 002 | C | LC437359.1 | A | MN996372.1 | neg | - | C/A |
| 003 | neg | - | A | MN996372.1 | neg | - | A |
| 004 | D | LC437360.1 | neg | - | neg | - | D |
| 005-010 | D | LC437360.1 | neg | - | neg | - | D |
| 011 | D | LC437360.1 | neg | - | neg | - | D |
| 013-017 | D | LC437360.1 | D | MT180093.1 | D | LC437463.1 | D |
| 036 | neg | - | A | MN996372.1 | neg | - | A |
| 054 | D | LC437360.1 | neg | - | neg | - | D |
| 055 | D | LC437360.1 | neg | - | neg | - | D |
| 056 | D | LC437360.1 | neg | - | neg | - | D |
| 057 | C | LC437359.1 | neg | - | neg | - | C |
| 058 | D | LC437360.1 | neg | - | neg | - | D |
| 059 | - | - | C | KY979489.1 | neg | - | C |
| 060 | neg | - | A | MN996372.1 | neg | - | A |
| 061 | A | MH047247.1 | A | MN996372.1 | neg | - | A |
| 062 | C | LC437359.1 | neg | - | neg | - | C |
| 063 | neg | - | A | MN996372.1 | neg | - | A |
| 065 | D | LC437360.1 | D | MF285574.1 | neg | - | D |
| 066 | D | LC437360.1 | neg | - | neg | - | D |
| 067 | neg | - | D | MF285574.1 | D | LC437447.1 | D |
| 068 | D | LC437360.1 | neg | - | neg | - | D |
| 070 | D | MK430923.1 | neg | - | neg | - | D |
| 071 | D | MK430923.1 | neg | - | neg | - | D |
| 072 | D | LC437360.1 | neg | - | D | LC437463.1 | D |
| 073 | C | LC437359.1 | D | MF285569.1 | neg | - | C/D |
| 074 | neg | - | D | MK968861.1 | neg | - | D |
| 076 | D | LC437360.1 | neg | - | neg | - | D |
| 080 | D | LC437360.1 | neg | - | neg | - | D |
| 082 | C | LC437359.1 | neg | - | neg | - | C |
| 083 | C | LC437359.1 | C | MF990015.1 | C | LC437435.1 | C |
| 085 | C | LC437359.1 | neg | - | neg | - | C |
| 087 | D | LC437360.1 | neg | - | neg | - | D |
| 089 | C | LC437359.1 | C | KY753393.1 | D | LC437463.1 | C/D |
| 093 | D | LC437360.1 | neg | - | neg | - | D |
| 096 | D | LC437360.1 | A | MN996372.1 | neg | - | D/A |
| 099 | C | LC437359.1 | neg | - | C | LC437440.1 | C |
| 100 | C | LC437359.1 | neg | - | C | LC437432.1 | C |
| 101 | neg | - | neg | - | C | LC437428.1 | C |
| 102 | C | LC437359.1 | C | KY979489.1 | C | LC437439.1 | C |
| 104 | D | LC437360.1 | neg | - | D | LC437450.1 | D |
| 105 | D | LC437360.1 | neg | - | neg | - | D |
| 106 | D | LC437360.1 | D | MK968861.1 | neg | - | D |
| 108 | D | LC437360.1 | D | MN270301.1 | D | LC437463.1 | D |
| 109 | - | - | C | KY753393.1 | C | LC437432.1 | C |
| 111 | C | LC437359.1 | neg | - | C | LC437432.1 | C |
| 115 | C | LC437359.1 | neg | - | neg | - | C |
| 124 | C | LC437359.1 | A | MN996372.1 | C | LC437435.1 | C/A |
| 132 | D | LC437360.1 | neg | - | D | LC437453.1 | D |
| 136 | C | LC437359.1 | neg | - | neg | - | C |
| 152 | C | LC437359.1 | neg | - | neg | - | C |
| 153 | C | LC437359.1 | neg | - | neg | - | C |
| 155 | C | LC437359.1 | neg | - | C | LC437432.1 | C |
| 156 | C | LC437359.1 | neg | - | neg | - | C |
| 158 | D | LC437360.1 | D | MF990017.1 | D | LC437463.1 | D |
| 159 | D | LC437360.1 | D | MF990017.1 | D | LC437463.1 | D |
| 190 | - | - | neg | - | neg | - | - |
| 191 | - | - | neg | - | neg | - | - |
| 210 | C | LC437359.1 | D | MF285574.1 | D | LC437458.1 | C/D |
| 211 | D | LC437360.1 | D | MK96886.1 | D | LC437458.1 | D |
| 217 | D | LC437360.1 | neg | - | neg | - | D |
| 218 | D | LC437360.1 | D | MF990017.1 | neg | - | D |
| 219 | C/D | LC437364.1 | D | MF990018.1 | D | LC437450.1 | C/D |
| 220 | C | LC437359.1 | neg | - | neg | - | C |
| 228 | D | LC437360.1 | neg | - | D | LC437463.1 | D |
| 236 | C | LC437359.1 | C | KY979489.1 | C | LC437432.1 | C |
| 244 | D | LC437360.1 | neg | - | neg | - | D |
| 249 | C | LC437359.1 | C | KY753393.1 | neg | - | C |
| 250 | C | LC437359.1 | C | KY979489.1 | C | LC437428.1 | C |
| 253 | C | LC437359.1 | neg | - | neg | - | C |
| 259 | C | LC437359.1 | neg | - | neg | - | C |
| 261 | C | LC437359.1 | neg | - | neg | - | C |
| 262 | - | - | C | KY979489.1 | neg | - | C |
| 264 | D | LC437360.1 | neg | - | neg | - | D |
| 279 | C | LC437359.1 | neg | - | neg | - | C |
| 304 | D | LC437360.1 | A | MN996372.1 | neg | - | D/A |
| 307 | - | - | neg | - | neg | - | - |
| 308 | D | LC437360.1 | neg | - | neg | - | D |
| 312 | C | LC437359.1 | C | KY979489.1 | C | LC437428.1 | C |
| 316 | C | LC437359.1 | neg | - | neg | - | C |
| 318 | C | LC437359.1 | neg | - | neg | - | C |
| 319 | - | - | neg | - | neg | - | - |
| 321 | C | LC437359.1 | neg | - | neg | - | C |
| 324 | D | LC437360.1 | neg | - | neg | - | D |
| 325 | C | LC437359.1 | neg | - | neg | - | C |
| 327 | D | LC437360.1 | D | MK968861.1 | neg | - | D |
| 328-335 | - | - | neg | - | neg | - | - |
| 342 | C | LC437359.1 | neg | - | C | LC437432.1 | C |
| 343 | neg | - | neg | - | C | LC437432.1 | C |
| 348 | C | LC437359.1 | neg | - | neg | - | C |
| 349 | D | LC437360.1 | D | MF285575.1 | D | LC437453.1 | D |
| 353 | neg | - | A | MN996372.1 | neg | - | A |
| 363 | neg | - | A | MN996372.1 | neg | - | A |
| 364 | C | LC437359.1 | neg | - | neg | - | C |
| 370 | C | LC437359.1 | neg | - | neg | - | C |
| 371 | C | LC437359.1 | neg | - | neg | - | C |
| 376 | C | LC437359.1 | neg | - | neg | - | C |
| 381 | C | LC437359.1 | neg | - | neg | - | C |
| 382 | C | LC437359.1 | neg | - | neg | - | C |
| 386 | C | LC437359.1 | D | MT180093.1 | neg | - | C/D |
| 388 | - | - | neg | - | neg | - | - |
| 389 | C | LC437359.1 | neg | - | neg | - | C |
| 391 | - |  | neg | - | neg | - | - |
| 395 | D | LC437360.1 | neg | - | neg | - | D |
| 398 | D | LC437360.1 | A | MN996372.1 | neg | - | D/A |
| 399 | - | - | neg | - | neg | - | - |
| 401 | D | LC437360.1 | neg | - | neg | - | D |
| 402 | D | LC437360.1 | neg | - | neg | - | D |
| 406 | C | LC437359.1 | A | MN996372.1 | neg | - | C/A |
| 413 | neg | - | neg | - | A | MK610391.1 | A |
| 416 | C | LC437359.1 | neg | - | neg | - | C |
| 417 | D | LC437360. | A | MN996372.1 | neg | - | D/A |
| 420 | A | LC437354.1 | A | MN996372.1 | neg | - | A |

neg= negative result

− = not evaluable
